# A phase 2b, randomized, double‐blind, multicenter, vehicle‐controlled study to assess the efficacy and safety of two crisaborole regimens in Japanese patients aged 2 years and older with mild‐to‐moderate atopic dermatitis

**DOI:** 10.1111/1346-8138.16120

**Published:** 2021-08-26

**Authors:** Kayo Fujita, Michio Yagi, Shinichi Moriwaki, Mizuki Yoshida, Daniela Graham

**Affiliations:** ^1^ Clinical Research Pfizer R&D Japan Tokyo Japan; ^2^ OPHAC Hospital Osaka Japan; ^3^ Department of Dermatology School of Medicine Osaka Medical and Pharmaceutical University Osaka Japan; ^4^ Clinical Statistics Pfizer R&D Japan Tokyo Japan; ^5^ Pfizer Inc. Groton Connecticut USA

**Keywords:** atopic dermatitis, clinical trial, Japan, pruritus, safety

## Abstract

Atopic dermatitis (AD) is a chronic inflammatory skin disease characterized by pruritus, xerosis, and eczematous lesions. In Japan, treatment options, such as topical corticosteroids and tacrolimus, are associated with efficacy and safety concerns. Crisaborole ointment, 2%, is a topical non‐steroidal anti‐inflammatory agent approved in several countries for the treatment of mild‐to‐moderate AD. This phase 2b, randomized, double‐blind study (NCT03954158) assessed the efficacy and safety of two crisaborole regimens versus vehicle in the treatment of Japanese patients aged ≥2 years with mild‐to‐moderate AD. Each patient was assigned to one of two age cohorts (≥12 or 2–11 years) and randomized to crisaborole once daily (QD) or twice daily (BID). All patients had two target lesions that were each randomly assigned to crisaborole or vehicle at baseline and treated for 2 weeks. The primary endpoint was change from baseline in total sign score (TSS) in crisaborole‐ or vehicle‐treated target lesions on day 15, and secondary endpoints included change from baseline in Investigator’s Static Global Assessment (ISGA) and pruritic assessments (Cohort 1: peak pruritus numeric rating scale [NRS]; Cohort 2: Itch Severity Scale Self‐Report and Caregiver‐Reported Itch Severity NRS) and incidence of treatment‐emergent adverse events (TEAEs). This study comprised 81 patients (Cohort 1: n = 41; Cohort 2: n = 40). Crisaborole‐treated lesions showed statistically significant reductions in TSS versus vehicle‐treated lesions at day 15 (*p* < 0.01), and numerically larger decreases in TSS were observed with crisaborole BID versus crisaborole QD in both cohorts. Furthermore, crisaborole‐treated lesions generally demonstrated greater decreases in ISGA, peak pruritus NRS, Itch Severity Scale, and Caregiver‐Reported Itch Severity NRS versus vehicle‐treated lesions irrespective of regimen or cohort. Overall, TEAEs were mild; the most frequently reported TEAEs was application site irritation. In summary, both crisaborole regimens, particularly crisaborole BID, demonstrated efficacy and were well tolerated.

## 1 INTRODUCTION

Atopic dermatitis (AD) is a chronic inflammatory skin disease characterized by pruritus, xerosis, and eczematous lesions that imposes a significant burden on patients.^1^, ^2^ The pathophysiological processes underlying AD include dysregulated inflammatory signaling and increased cytokine production compared with normal skin.^1^ The prevalence of AD has varied worldwide;^3^, ^4^ however, the prevalence of AD has increased in certain regions, particularly in the Asia‐Pacific region.^4^, ^5^ Findings from a recent cross‐sectional survey‐based study suggest that the burden of AD is substantially high in Japanese adults, with direct medical costs estimated at ¥450 billion/year.^2^

Current treatment guidelines recommend topical corticosteroids or tacrolimus for the initial treatment and management of AD in Japan.^6^ However, topical corticosteroids are associated with local side‐effects, including skin atrophy and increased risk of skin infection, and tacrolimus may demonstrate limited efficacy.^6^ Delgocitinib is a topical Janus kinase inhibitor that was recently approved for the treatment of adult and pediatric patients with AD in Japan;^7^, ^8^ however, skin infections, such as application site folliculitis and Kaposi varicelliform eruption, have been associated with delgocitinib use.^9^ Therefore, an unmet need exists for additional treatment options with improved efficacy and safety profiles.^7^

Crisaborole ointment, 2%, is a non‐steroidal anti‐inflammatory phosphodiesterase 4 (PDE4) inhibitor approved for twice daily (BID) use in patients aged ≥2 years with mild‐to‐moderate AD in several regions, including Australia, Canada, the EU, and Israel.^10^, ^11^, ^12^, ^13^ In Lebanon and the USA, crisaborole is approved for patients as young as 3 months of age.^14^, ^15^ Regulatory approval was based on two identically designed, vehicle‐controlled, randomized, double‐blind phase 3 studies (CrisADe CORE 1 and CORE 2) that assessed the efficacy and safety of crisaborole treatment in patients aged ≥2 years with mild‐to‐moderate AD.^16^ The proportion of patients who achieved the primary endpoint of Investigator’s Static Global Assessment (ISGA) success (score of clear [0] or almost clear [1] with a ≥2‐grade improvement from baseline) with crisaborole was statistically significant compared with vehicle at day 29 (CORE 1: 32.8% vs 25.4%, *p* = 0.038; CORE 2: 31.4% vs 18.0%, *p* < 0.001), and crisaborole treatment was well tolerated.^16^ Crisaborole also demonstrated a favorable safety profile in an open‐label, 48‐week extension study of patients with mild‐to‐moderate AD (n = 517).^17^

The safety and pharmacokinetics of crisaborole treatment were recently evaluated in a phase 1 study with healthy Japanese subjects and Japanese patients with mild‐to‐moderate AD.^18^ Crisaborole was generally well tolerated in both populations, and the pharmacokinetic profile of crisaborole in patients with mild‐to‐moderate AD was consistent with that of US‐based patients.^18^ To extend these findings, a phase 2b, randomized, double‐blind, vehicle‐controlled study was conducted to evaluate the efficacy and safety of two crisaborole treatment regimens in Japanese pediatric and adult patients with mild‐to‐moderate AD.

## 2 METHODS

### 2.1 Study design

This was a phase 2b, randomized, multicenter, double‐blind, vehicle‐controlled intrapatient study (NCT03954158) that was conducted at three study sites from June to December 2019. Following screening, two target lesions that were moderate in severity were identified on each patient. These lesions were ≥10 cm apart and ≥3 cm × 3 cm with ISGA = 3 (moderate). At baseline (day 1), patients in each age cohort (Cohort 1: ≥12 years; Cohort 2: 2–11 years) were randomized via interactive response technology to once daily (QD) or BID regimens, and crisaborole 2% or vehicle was randomly assigned to each target lesion at baseline (day 1) and administrated for a 2‐week period. Patient follow‐up was 28 days following the end of the treatment period.

The final protocol and informed consent/assent were reviewed and approved by the institutional review board at each investigational center participating in this study. This study was conducted in compliance with the ethical principles of the Declaration of Helsinki and in compliance with the International Committee on Harmonisation and Good Clinical Practice Guidelines, and all local regulatory requirements were followed. Informed consent was provided by patients or parent(s)/guardian(s) of pediatric patients.

### 2.2 Patients and treatment

All patients had a confirmed clinical diagnosis of active AD at screening and baseline (day 1) according to Hanifin and Rajka criteria^19^ and ≥6 months history of AD prior to screening. In addition, AD had been clinically stable for >1 month with an ISGA of 2 (mild) or 3 (moderate) at baseline (day 1). Patients with a previous history of angioedema, anaphylaxis, sensitivity to any component of crisaborole ointment, or treatment with any topical or systemic PDE4 inhibitor were excluded from this study. Patients had AD lesions on their upper or lower limbs or ventral body trunk and a percentage of treatable body surface area (%BSA) of 1% but not >30% at baseline, excluding the scalp, genitals, and groin area. When possible, AD lesions on bilateral areas were selected as target lesions; two AD lesions on the same limb were not selected as target lesions. Selected target lesions were not located on the face, neck, scalp, axilla, genitals, groin area, palms, dorsal side of hands, dorsal side of the body trunk, or soles. Target lesions were not expected to exceed 30% BSA. Crisaborole, 2%, ointment or vehicle ointment was applied topically as a thin layer to each target lesion during the 2‐week study period. Crisaborole 2% or vehicle ointment was applied using the fingertip unit, and the amount of ointment applied to target lesions was dependent on the target lesion area and determined by the principal investigator. Target lesion area was calculated using the handprint method, in which the area represented by the participant’s outstretched hand was equal to approximately 1% of the participant’s BSA. Crisaborole 2% and vehicle were not applied to other AD‐affected areas; however, patients were permitted to use emollients, moisturizers, topical corticosteroids, and topical calcineurin inhibitors if applied ≥10 cm away from selected target lesions. The application of ointment was always performed under supervision (either applied directly or supervised by study personnel) in both cohorts.

### 2.3 Assessments

The primary endpoint was the change from baseline in total sign score (TSS) in target lesions treated with crisaborole or vehicle on day 15 for each regimen in each cohort. The TSS evaluated the severity of erythema, induration/papulation, excoriation, and lichenification using a 4‐point severity scale; the sum of these ratings generated a total score on a 13‐point scale (0–12 points).

Secondary endpoints included the following: change from baseline in TSS in target lesions treated with crisaborole 2% QD or BID on day 8 and day 15 for each cohort; change from baseline in ISGA, a 5‐point clinician‐reported scale assessing AD severity on a 0–4 scale, with “0” corresponding to “clear” and “4” corresponding to “severe”,^16^ in target lesions treated with crisaborole 2% (QD or BID) or vehicle at days 8 and 15 for each cohort; change from baseline in pruritic assessments in target lesions treated with crisaborole 2% or vehicle up to day 15 for each regimen as measured by peak pruritus numeric rating scale (NRS)^20^ in Cohort 1 and the Itch Severity Scale Self‐Report (patients aged 6–11 years) and Caregiver‐Reported Itch Severity NRS (patients aged 2–11 years) in Cohort 2. Peak pruritus and caregiver‐reported itch severity were evaluated using 11‐point scales, whereas itch severity was evaluated on a 5‐point scale. Safety of both crisaborole regimens was assessed via TEAEs (all cause and treatment related) and serious adverse events (SAEs); only TEAEs occurring in the target lesion were considered treatment related. TEAEs and SAEs were coded using the Medical Dictionary for Regulatory Activities version 22.1.

### 2.4 Statistical analysis

The full analysis set (FAS) comprised all randomized patients who received ≥1 dose of study drug, and the per‐protocol analysis set (PPAS) consisted of all randomized patients who received ≥1 dose of study drug, with both baseline and day 15 primary efficacy data, and without protocol violations that may have impacted the efficacy evaluation during the treatment period. The safety analysis set (SAF) comprised all patients who received ≥1 dose of study drug. Efficacy analyses were performed in the FAS and PPAS, and safety analyses were performed in the SAF. The main analyses of TSS, ISGA, and pruritic assessments were based on the FAS. Baseline was defined as the last observation up to and including the first dosing date.

Analysis of efficacy parameters (TSS, ISGA, peak pruritus NRS [Cohort 1 only], Itch Severity Scale Self‐Report [Cohort 2 only], and Caregiver‐Reported Itch Severity NRS [Cohort 2 only]) was based on intrapatient and interpatient comparisons. For intrapatient comparisons of crisaborole versus vehicle in each regimen for each cohort, mixed‐effect models for repeated measures (MMRM) was used to derive the least squares mean (LSM) of intrapatient difference and associated two‐sided 95% confidence interval (CI). MMRM included the fixed effect of time point and an unstructured variance and covariance matrix to model the dependence among the same patients across different visits up to day 15. When there was a convergence issue for unstructured variance and the covariance matrix, first‐order autoregressive variance and the covariance matrix were used. For interpatient comparisons of crisaborole 2% BID versus QD in each cohort, MMRM was used to calculate the LSM difference and associated two‐sided 95% CI between crisaborole 2% BID and QD. MMRM included the fixed effects of dosing regimen, time point, dosing regimen‐by‐time point interaction and baseline value of the corresponding endpoint, and an unstructured variance and covariance matrix to model the dependence among the same patients across different visits up to day 15. An MMRM analysis was conducted that included only the observed data in the model under the assumption of missing at random for the missing mechanism.

For the main analysis of the primary endpoint for each regimen in each cohort, crisaborole 2% was superior to vehicle if the change in TSS for crisaborole treatment was greater than that for vehicle and *p* < 0.05 (2‐sided). A sample size of 16 would have had ≥85% power to establish the superiority of crisaborole 2% to vehicle as measured by change from baseline in TSS between crisaborole‐ and vehicle‐treated lesions at day 15 using a paired *t*‐test at 0.025 (one‐sided) significance level, assuming the mean of intrapatient difference was 1.8 and the standard deviation (SD) was 2.2. In contrast to the main analysis of the primary endpoint, *p*‐values for secondary endpoints were considered to be nominal as no adjustments for multiplicity were conducted.

Safety data were presented using descriptive statistics. Statistical analyses were conducted using SAS version 9.4 (SAS Institute, Cary, NC, USA).

## 3 RESULTS

### 3.1 Baseline characteristics

Eighty‐one patients (Cohort 1: n = 41; Cohort 2: n = 40) comprised the FAS, PPAS, and SAF, and all patients completed the study. No patients were excluded from the FAS or PPAS; therefore, the FAS and PPAS populations were identical. Demographic and baseline disease characteristics for each cohort are shown in Table 1. In Cohort 1, the mean age (SD) was 33.3 (10.4) and 33.9 (11.0) years in the crisaborole 2% QD and BID groups, respectively. In Cohort 2, the mean age (SD) was 7.7 (2.5) and 7.8 (2.7) years in the crisaborole 2% QD and BID groups, respectively. TSS was balanced in target lesions between assigned treatments in each regimen for each cohort (Table S1).

**TABLE 1:** Demographic and baseline characteristics (full analysis set)

	Cohort 1 (aged ≥12 years)		Cohort 2 (aged 2–11 years)	
	Crisaborole 2% QD + vehicle QD n = 20	Crisaborole 2% BID + vehicle BID n = 21	Crisaborole 2% QD + vehicle QD n = 20	Crisaborole 2% BID + vehicle BID n = 20
Age, years^a^				
Mean (SD)	33.3 (10.4)	33.9 (11.0)	7.7 (2.5)	7.8 (2.7)
Median (range)	33.5 (18–52)	33.0 (21–55)	7.5 (3–11)	8.5 (2–11)
Sex, n (%)				
Male	11 (55.0)	17 (81.0)	8 (40.0)	11 (55.0)
Female	9 (45.0)	4 (19.0)	12 (60.0)	9 (45.0)
Treatable %BSA (global), %				
Mean (SD)	18.5 (7.4)	18.3 (7.2)	4.8 (2.3)	9.2 (7.6)
Median (range)	21.3 (3.3–30.0)	18.0 (6.5–30.0)	4.4 (1.7–10.5)	6.9 (2.9–29.3)
ISGA (global)				
Mean (SD)	2.4 (0.5)	2.7 (0.5)	2.2 (0.4)	2.8 (0.4)
Median (range)	2.0 (2–3)	3.0 (2–3)	2.0 (2–3)	3.0 (2–3)

Abbreviations: BID, twice daily; %BSA, percentage of body surface area; ISGA, Investigator’s Static Global Assessment; QD, once daily; SD, standard deviation.

^a^ Age at screening (years) = (date of given informed consent) – (date of birth + 1) / 365.25.

### 3.2 Efficacy

#### 3.2.1 Change from baseline in TSS

**FIGURE 1:**
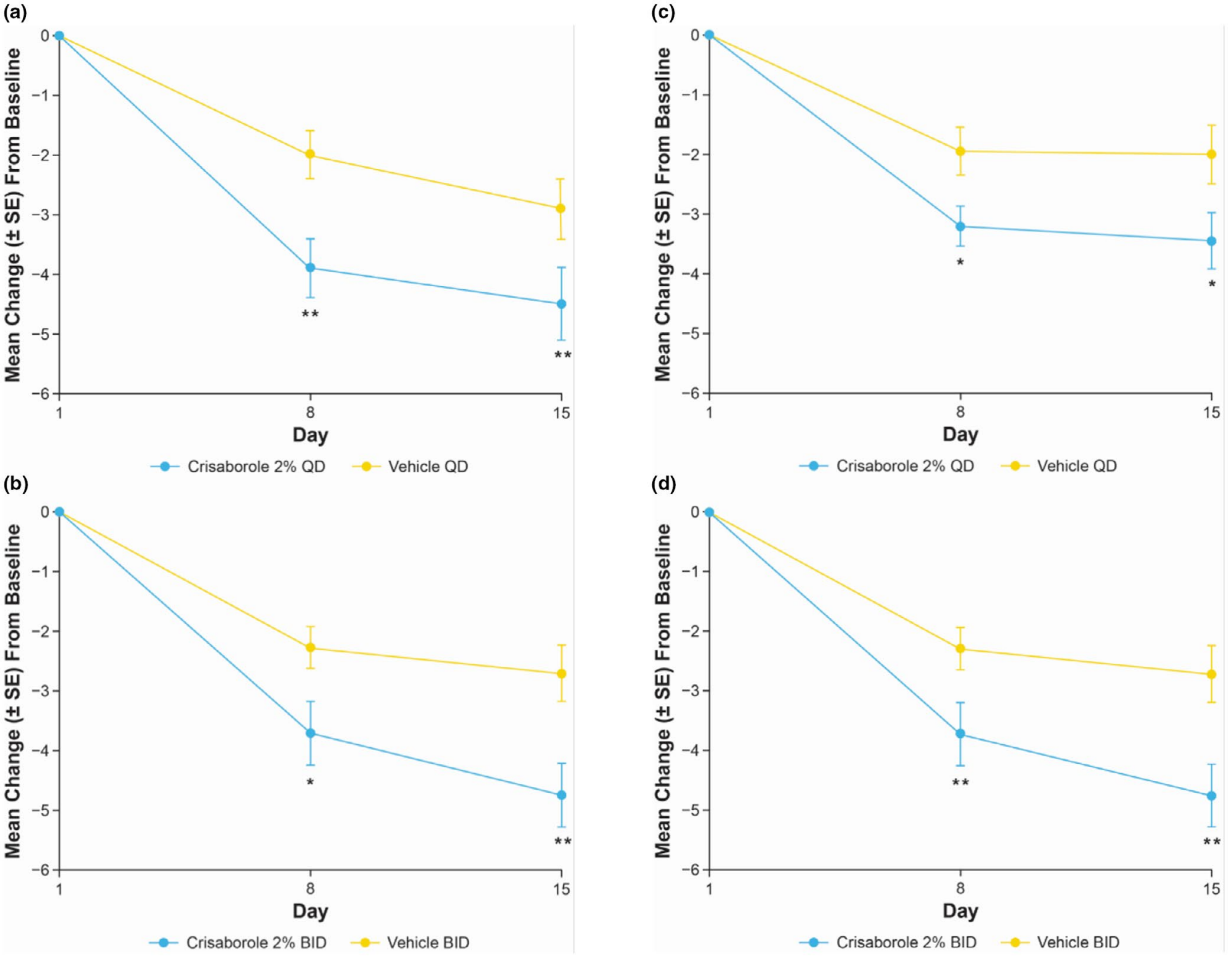
Mean change (SE) from baseline to day 8 and day 15 in TSS for intrapatient comparison of crisaborole 2% QD and BID versus vehicle in (a,b) Cohort 1 and (c,d) Cohort 2 (full analysis set).^†,‡^ *p < 0.05 and **p < 0.01 for intrapatient comparison of crisaborole 2% vs vehicle based on MMRM. ^†^MMRM includes the fixed effect of visit, and an unstructured covariance structure was used. ^‡^Adjustments for multiplicity were not conducted at day 8. BID, twice daily; MMRM, mixed‐effect model for repeated measures; QD, once daily; SE, standard error; TSS, total sign score

In Cohort 1, statistically significant decreases in target lesion TSS were observed with crisaborole 2% QD and BID compared with vehicle at day 15 (Figure 1a,b). The LSM of intrapatient difference (95% CI) of TSS change from baseline for crisaborole 2% QD and BID was −1.6 (95% CI, −2.7 to −0.5; *p* = 0.0071) and −2.0 (95% CI, −3.3 to −0.8; *p* = 0.0029), respectively. Similarly, statistically significant decreases in target lesion TSS were observed with crisaborole 2% QD and BID compared with vehicle at day 15 in Cohort 2 (Figure 1c,d). The LSM of intrapatient difference (95% CI) of TSS change from baseline for crisaborole 2% QD and BID was −1.5 (95% CI, −2.7 to −0.2; *p* = 0.0250) and −2.1 (95% CI, −3.3 to −0.9; *p* = 0.0014), respectively.

**FIGURE 2:**
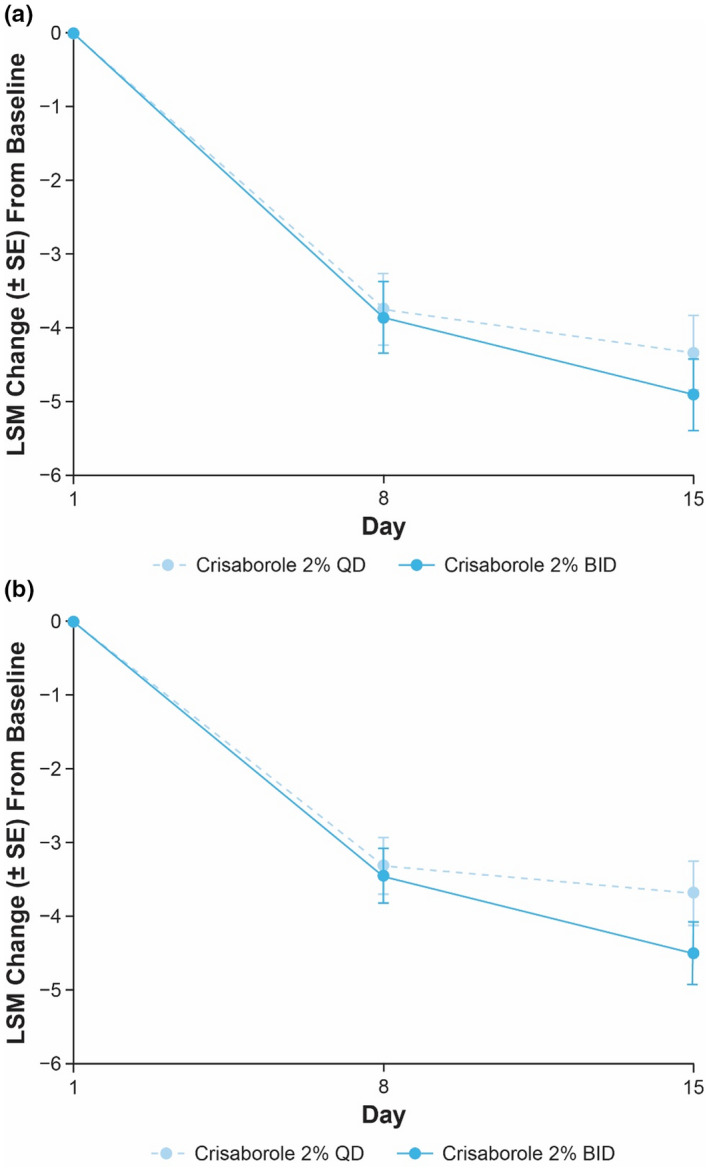
LSM change (SE) from baseline to day 8 and day 15 in TSS for patients receiving crisaborole 2% QD or crisaborole 2% BID in (a) Cohort 1 and (b) Cohort 2 (full analysis set).^†,‡ †^Nominal *p* ≥ 0.05 for interpatient comparisons of crisaborole 2% BID vs crisaborole 2% QD in Cohort 1 and Cohort 2 based on MMRM at days 8 and 15. ^‡^MMRM includes the fixed effects of dosing regimen, visit, dosing regimen‐by‐visit interaction, and baseline value, and an unstructured covariance structure was used. BID, twice daily; LSM, least squares mean; MMRM, mixed‐effect model for repeated measures; QD, once daily; SE, standard error; TSS, total sign score

The decrease in TSS at day 15 for target lesions treated with crisaborole 2% BID was numerically greater than that observed for target lesions treated with crisaborole 2% QD in both cohorts (Figure 2). The LSM interpatient difference (95% CI) of TSS change from baseline between both crisaborole regimens was −0.6 (−2.0 to 0.9) and −0.8 (−2.1 to 0.4) for Cohort 1 and Cohort 2, respectively.

At day 8, LSM of intrapatient difference (95% CI) for crisaborole 2% QD‐ and BID‐treated target lesions compared with vehicle‐treated lesions was −1.9 (−3.1 to −0.7) and −1.4 (−2.7 to −0.2), respectively, in Cohort 1; however, the LSM interpatient difference (95% CI) of TSS change from baseline between crisaborole 2% BID and QD was negligible at this time point (−0.1 [−1.5 to 1.3]). In Cohort 2, LSM of intrapatient difference (95% CI) of TSS change from baseline with crisaborole 2% QD and BID compared with vehicle was −1.2 (−2.2 to −0.2) and −1.7 (−2.8 to −0.5), respectively, at day 8. In accordance with the observations in Cohort 1, the LSM interpatient difference (95% CI) of TSS change from baseline of crisaborole 2% BID and QD was negligible at day 8 (−0.1 [−1.2 to 1.0]).

#### 3.2.2 Change from baseline in ISGA

In Cohort 1, the LSM of intrapatient difference (95% CI) of change from baseline in ISGA for crisaborole 2% QD and BID compared with vehicle at day 15 was −0.9 (−1.4 to −0.4) and −0.7 (−1.2 to −0.2), respectively. The difference of LSM of change from baseline (95% CI) between crisaborole 2% BID and QD was 0 (−0.5 to 0.6) at day 15 (Table 2). Similar results were observed at day 8.

**TABLE 2:** Change from baseline in ISGA at day 8 and day 15 in Japanese patients receiving crisaborole 2% or vehicle as assessed by MMRM (full analysis set)

	Cohort 1 (aged ≥12 years)				Cohort 2 (aged 2–11 years)			
	QD regimen (n = 20)		BID regimen (n = 21)		QD regimen (n = 20)		BID regimen (n = 20)	
	Crisaborole 2%	Vehicle	Crisaborole 2%	Vehicle	Crisaborole 2%	Vehicle	Crisaborole 2%	Vehicle
Intrapatient comparison between crisaborole and vehicle^a^								
Mean change (SE) from baseline (day 8)	−1.3 (0.2)	−0.8 (0.1)	−1.1 (0.2)	−0.7 (0.1)	−1.4 (0.2)	−1.0 (0.2)	−1.2 (0.2)	−0.8 (0.2)
LSM of intrapatient difference (95% CI) (crisaborole vs vehicle)	−0.5 (−0.9 to −0.1)		−0.4 (−0.9 to 0)		−0.3 (−0.8 to 0.1)		−0.4 (−0.8 to 0)	
Mean change (SE) from baseline (day 15)	−1.9 (0.2)	−1.0 (0.2)	−1.9 (0.2)	−1.1 (0.2)	−1.8 (0.2)	−1.1 (0.2)	−1.9 (0.2)	−0.9 (0.2)
LSM of intrapatient difference (95% CI) (crisaborole vs vehicle)	−0.9 (−1.4 to −0.4)		−0.7 (−1.2 to −0.2)		−0.7 (−1.2 to −0.2)		−1.0 (−1.4 to −0.6)	
Interpatient comparison between crisaborole BID and QD^b^								
LSM of change from baseline (95% CI) (day 8)	−1.3 (−1.6 to −0.9)	–	−1.1 (−1.5 to −0.8)	–	−1.3 (−1.7 to −1.0)	–	−1.2 (−1.5 to −0.8)	–
Difference of LSM of change from baseline (95% CI) between crisaborole BID and QD	0.1 (−0.4 to 0.6)				0.2 (−0.3 to 0.7)			
LSM of change from baseline (95% CI) (day 15)	−1.9 (−2.3 to −1.5)	–	−1.9 (−2.2 to −1.5)	–	−1.8 (−2.2 to −1.4)	–	−1.9 (−2.3 to −1.5)	–
Difference of LSM of change from baseline (95% CI) between crisaborole BID and QD	0 (−0.5 to 0.6)				−0.1 (−0.7 to 0.5)			

Abbreviations: BID, twice daily; CI, confidence interval; ISGA, Investigator’s Static Global Assessment; LSM, least squares mean; MMRM, mixed‐effect model for repeated measures; QD, once daily; SE, standard error.

^a^ MMRM includes the fixed effect of visit, and an unstructured covariance structure is used.

^b^ MMRM includes the fixed effects of dosing regimen, visit, dosing regimen‐by‐visit interaction, and baseline value, and an unstructured covariance structure is used.

In Cohort 2, the LSM of intrapatient difference (95% CI) of change from baseline in ISGA for crisaborole 2% QD and BID compared with vehicle at day 15 was −0.7 (−1.2 to −0.2) and −1.0 (−1.4 to −0.6), respectively. The difference of LSM of change from baseline (95% CI) between crisaborole 2% BID and QD was −0.1 (−0.7 to 0.5) at day 15 (Table 2). Similar results were observed at day 8.

#### 3.2.3 Change from baseline in peak pruritus NRS, Itch Severity Scale, and Caregiver‐Reported Itch Severity severity NRS

**FIGURE 3:**
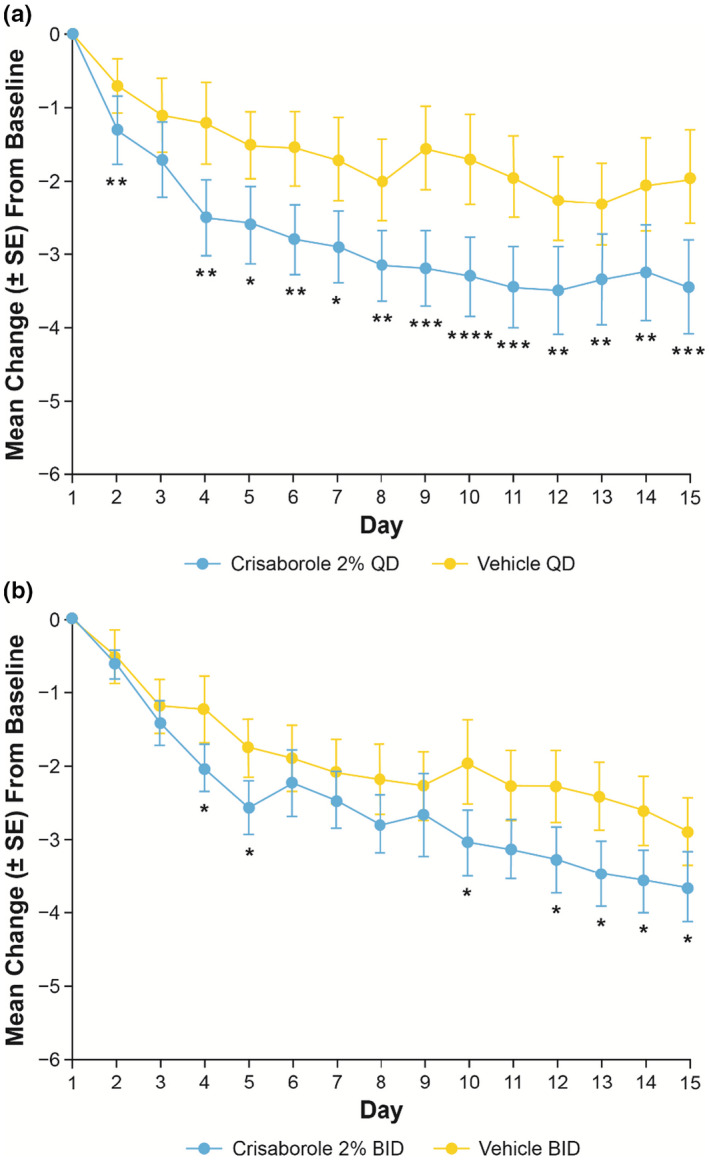
Mean change (SE) from baseline up to day 15 in peak pruritus NRS for patients aged ≥12 years (Cohort 1) receiving (a) crisaborole 2% QD or (b) crisaborole 2% BID versus vehicle (full analysis set).^†,‡ †^Nominal *p < 0.05, **p < 0.01, ***p < 0.001, and ****p < 0.0001 for intrapatient comparison of crisaborole 2% vs vehicle based on MMRM. ^‡^MMRM includes the fixed effect of visit, and an unstructured covariance structure was used. BID, twice daily; MMRM, mixed‐effect model for repeated measures; NRS, numeric rating scale; QD, once daily; SE, standard error

**FIGURE 4:**
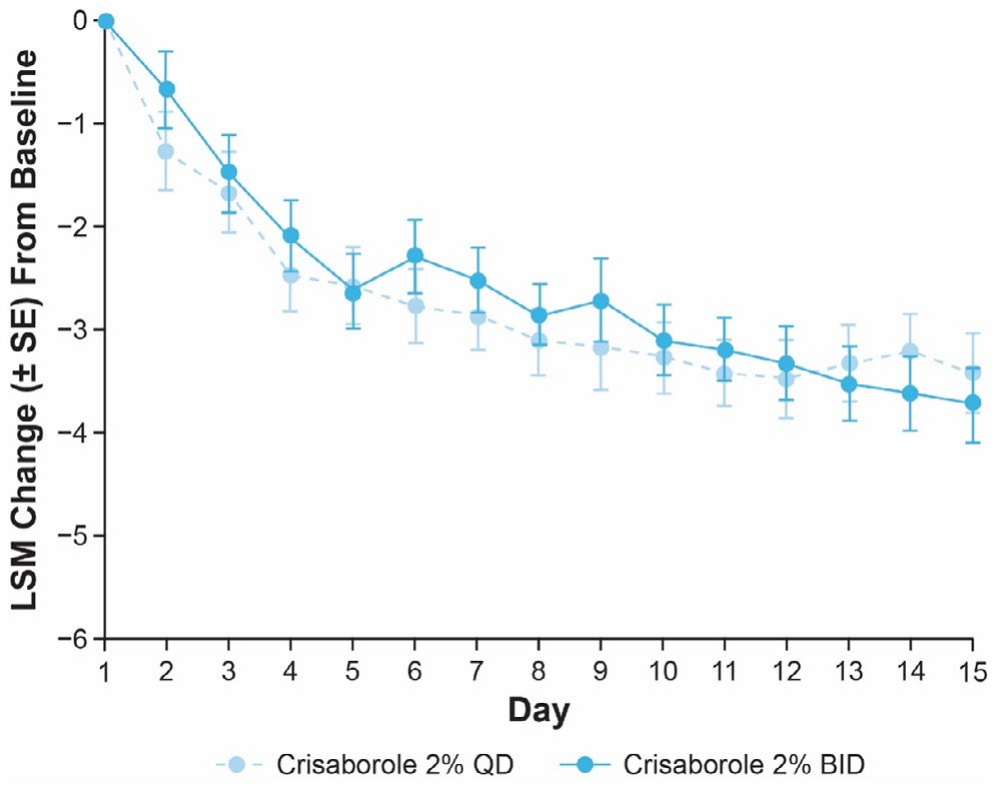
LSM change (SE) from baseline up to day 15 in peak pruritus NRS for patients aged ≥12 years (Cohort 1) receiving crisaborole 2% QD or crisaborole 2% BID (full analysis set). ^†,‡ †^Nominal *p* ≥ 0.05 for interpatient comparisons of crisaborole 2% BID versus crisaborole 2% QD based on MMRM from baseline to day 15. ^‡^MMRM includes the fixed effects of dosing regimen, day, dosing regimen‐by‐day interaction, and baseline value, and an unstructured covariance structure was used. BID, twice daily; LSM, least squares mean; MMRM, mixed‐effect model for repeated measures; NRS, numeric rating scale; QD, once daily; SE, standard error

In Cohort 1, target lesions treated with crisaborole 2% QD and BID demonstrated greater improvements in peak pruritus NRS compared with those treated with vehicle on the day following the first application (crisaborole 2% QD mean change [standard error [SE]]: −1.3 [0.5]; vehicle QD: −0.7 [0.4]; crisaborole 2% BID: −0.6 [0.2]; vehicle BID: −0.5 [0.4]) (Figure 3). Improvements were maintained through day 15 (crisaborole 2% QD: −3.5 [0.6]; vehicle QD: −2.0 [0.6]; crisaborole 2% BID: −3.7 [0.5]; vehicle BID: −2.9 [0.5]). The LSM change from baseline in peak pruritus NRS was generally greater in patients treated with crisaborole 2% QD compared with crisaborole 2% BID from day 2 to day 12; however, the LSM change from baseline was greater with crisaborole 2% BID compared with crisaborole 2% QD after day 13 (Figure 4).

**FIGURE 5:**
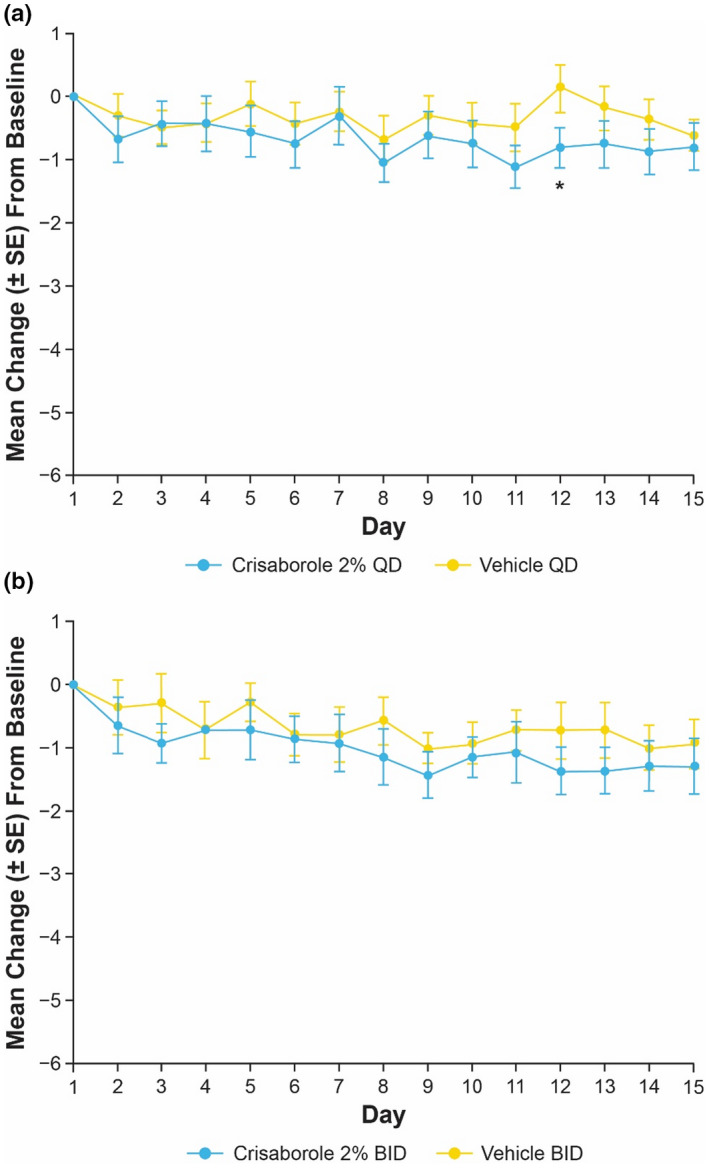
Mean change (SE) from baseline up to day 15 in Itch Severity Scale for patients aged 6–11 years (Cohort 2) receiving (a) crisaborole 2% QD or (b) crisaborole 2% BID versus vehicle (full analysis set).^†,‡ †^Nominal *p < 0.05 for intrapatient comparison of crisaborole 2% versus vehicle based on MMRM. ^‡^MMRM includes the fixed effect of visit, and an unstructured covariance structure for (a) and first‐order autoregressive covariance structure for (b) were used. BID, twice daily; MMRM, mixed‐effect model for repeated measures; QD, once daily; SE, standard error

**FIGURE 6:**
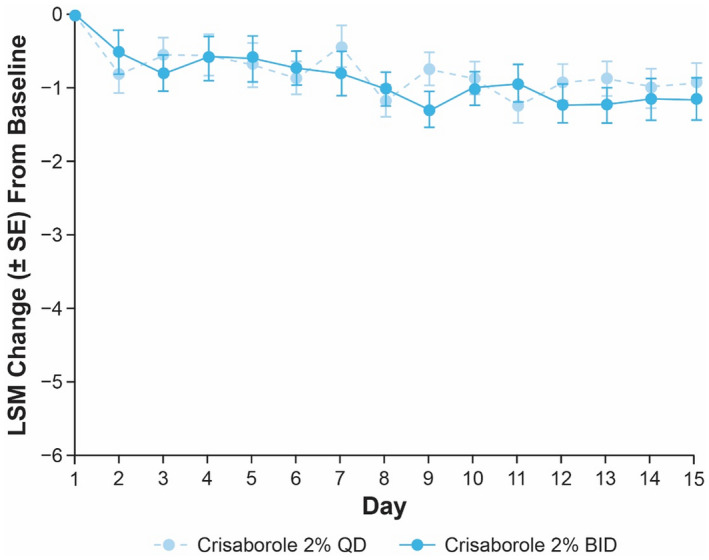
LSM change (SE) from baseline up to day 15 in Itch Severity Scale for patients aged 6–11 years (Cohort 2) receiving crisaborole 2% QD or crisaborole 2% BID (full analysis set).^†,‡ †^Nominal *p* ≥ 0.05 for interpatient comparisons of crisaborole 2% BID versus crisaborole 2% QD based on MMRM from baseline to day 15. ^‡^MMRM includes the fixed effects of dosing regimen, day, dosing regimen‐by‐day interaction, and baseline value, and an unstructured covariance structure was used. BID, twice daily; LSM, least squares mean; MMRM, mixed‐effect model for repeated measures; QD, once daily; SE, standard error

In Cohort 2, target lesions treated with crisaborole 2% QD or BID in patients aged 6–11 years demonstrated improvement in the Itch Severity Scale with higher frequency than those treated with vehicle on the day following the first application (crisaborole 2% QD mean change [SE]: −0.7 [0.4]; vehicle QD: −0.3 [0.4]; crisaborole 2% BID: −0.6 [0.5]; vehicle BID: −0.4 [0.4]) (Figure 5). At day 15, the mean change (SE) in Itch Severity Scale with crisaborole 2% QD and vehicle QD was −0.8 (0.4) and −0.6 (0.3), respectively, and −1.3 (0.4) and −0.9 (0.4) with crisaborole 2% BID and vehicle BID, respectively. Among patients aged 6–11 years in Cohort 2, the LSM change from baseline with crisaborole 2% QD versus BID was generally comparable from day 2 to day 15 (Figure 6).

**FIGURE 7:**
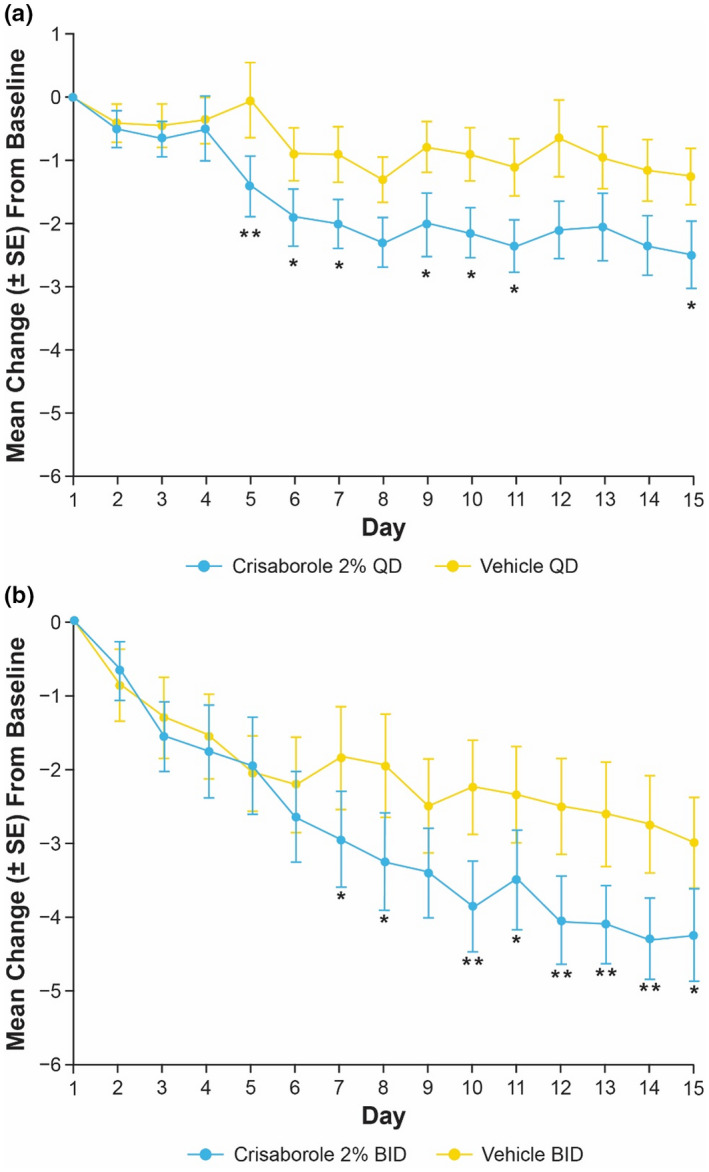
Mean change (SE) from baseline up to day 15 in Caregiver‐Reported Itch Severity NRS for patients aged 2–11 years (Cohort 2) receiving (a) crisaborole 2% QD or (b) crisaborole 2% BID versus vehicle (full analysis set).^†,‡ †^Nominal *p < 0.05 and **p < 0.01 for intrapatient comparison of crisaborole 2% versus vehicle based on MMRM. ^‡^MMRM includes the fixed effect of visit, and an unstructured covariance structure was used. BID, twice daily; MMRM, mixed‐effect model for repeated measures; NRS, numeric rating scale; QD, once daily; SE, standard error

**FIGURE 8:**
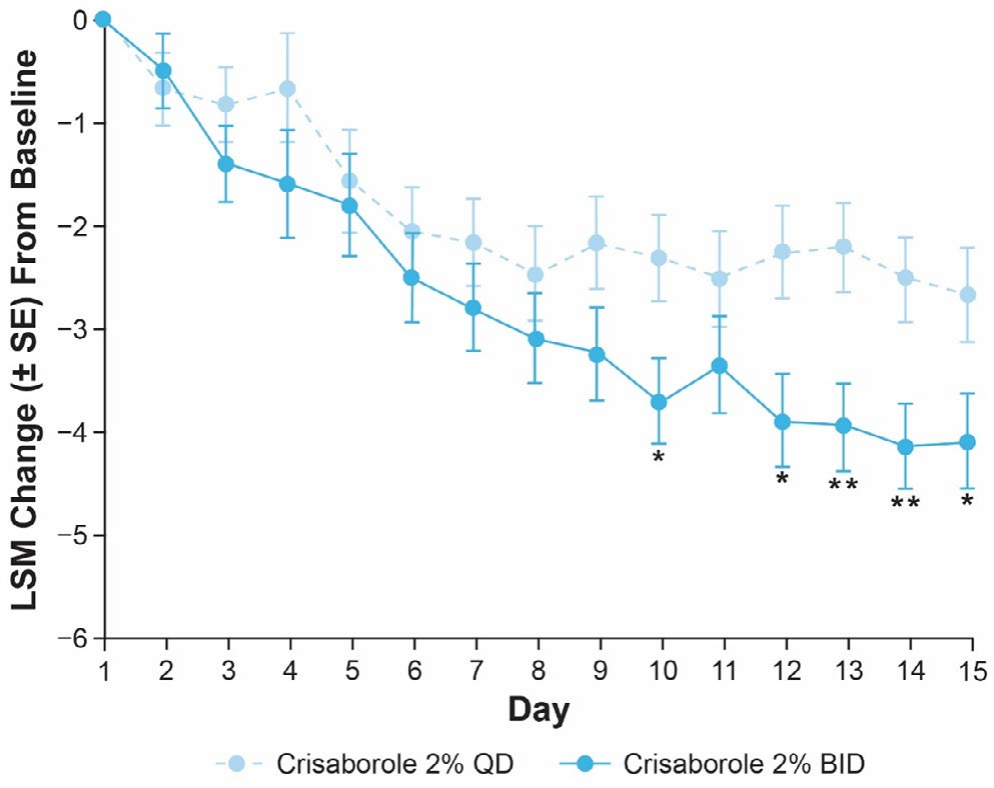
LSM change (SE) from baseline up to day 15 in Caregiver‐Reported Itch Severity NRS for patients aged 2–11 years (Cohort 2) receiving crisaborole 2% QD or crisaborole 2% BID (full analysis set).^†,‡ †^Nominal *p < 0.05 and **p < 0.01 for interpatient comparison of crisaborole 2% BID versus crisaborole 2% QD based on MMRM. ^‡^MMRM includes the fixed effects of dosing regimen, day, dosing regimen‐by‐day interaction, and baseline value, and an unstructured covariance structure was used. BID, twice daily; LSM, least squares mean; MMRM, mixed‐effect model for repeated measures; NRS, numeric rating scale; QD, once daily; SE, standard error

Among patients aged 2–11 years in Cohort 2, a greater decrease from baseline in Caregiver‐Reported Itch Severity NRS was observed with crisaborole 2% QD compared with vehicle on the day following the first application (crisaborole 2% QD mean change [SE]: −0.5 [0.3]; vehicle QD: −0.4 [0.3]), and improvement was maintained through day 15 (crisaborole 2% QD: −2.5 [0.6]; vehicle QD: −1.3 [0.5]) (Figure 7a). With crisaborole 2% BID, less improvement was observed compared with vehicle on the day following the first application (crisaborole 2% BID mean change [SE]: −0.7 [0.4]; vehicle BID: −0.9 [0.5]) but a greater decrease in Caregiver‐Reported Itch Severity Scale NRS was observed at day 15 (crisaborole 2% BID: −4.3 [0.6]; vehicle BID: −3.0 [0.6]) (Figure 7b). In patients aged 2–11 years in Cohort 2, the LSM change from baseline in Caregiver‐Reported Itch Severity Scale was generally greater with crisaborole 2% BID compared with crisaborole 2% QD from day 2 to day 15 (Figure 8).

### 3.3 Safety

In Cohort 1, the median (range) duration of treatment with both crisaborole regimens or vehicle was 14 (14–15) days, and the median (range) number of crisaborole/vehicle applications was 14 (14–15) and 28 (28–30) with crisaborole 2% QD and BID, respectively. In Cohort 2, the median (range) duration of treatment with crisaborole 2% QD or vehicle was 14 (14–16) days, and the median (range) duration of treatment with crisaborole 2% BID or vehicle was 14 (14–17) days. The median (range) number of crisaborole/vehicle applications was 14 (14–16) and 28 (28–34) applications with crisaborole 2% QD and BID, respectively. All applications of ointment were performed by study personnel (Cohort 1) or confirmed by study personnel (Cohort 2), and all patients in both regimens were considered compliant with treatment except one patient receiving crisaborole 2% BID in Cohort 2. There were no SAEs, dose reductions, or temporary/permanent discontinuations due to TEAEs in this study, and all TEAEs were mild in severity.

TEAEs by system organ class and preferred term are presented in Table 3. In Cohort 1, six (30.0%) patients receiving crisaborole 2% QD and six (28.6%) patients receiving crisaborole 2% BID experienced TEAEs. The most frequently reported TEAEs with crisaborole 2% QD were application site irritation and application site pruritus (each n = 3), all of which were treatment related. The most frequently reported TEAEs with crisaborole 2% BID were application site irritation (n = 4) and oropharyngeal pain (n = 3); only application site irritation was considered to be related to crisaborole treatment. In Cohort 2, two (10.0%) patients receiving crisaborole 2% QD and two (10.0%) patients receiving crisaborole 2% BID experienced TEAEs. Reported TEAEs were arthralgia and hand, foot, and mouth disease (each n = 1) with crisaborole 2% QD and application site pruritus, application site pain, and hand, foot, and mouth disease (each n = 1) with crisaborole 2% BID. Of these TEAEs, none were related to crisaborole 2% QD, whereas application site pruritus and application site pain were considered to be related to crisaborole 2% BID.

**TABLE 3:** TEAEs by System Organ Class and Preferred Term in Japanese pediatric and adult patients receiving crisaborole 2% or vehicle^a^ (safety analysis set)

n (%)	Cohort 1 (aged ≥12 years)		Cohort 2 (aged 2–11 years)	
	Crisaborole 2% QD + vehicle QD n = 20	Crisaborole 2% BID + vehicle BID n = 21	Crisaborole 2% QD +vehicle QD n = 20	Crisaborole 2% BID + vehicle BID n = 20
Any adverse event	6 (30.0)	6 (28.6)	2 (10.0)	2 (10.0)
Gastrointestinal disorders	1 (5.0)	0	0	0
Dental caries	1 (5.0)	0	0	0
general disorders and administration site conditions	5 (25.0)	5 (23.8)	0	1 (5.0)
Application site coldness	0	1 (4.8)	0	0
Application site irritation	3 (15.0)	4 (19.0)	0	0
Application site pain	1 (5.0)	1 (4.8)	0	1 (5.0)
Application site pruritus	3 (15.0)	2 (9.5)	0	1 (5.0)
Infections and infestations	1 (5.0)	1 (4.8)	1 (5.0)	1 (5.0)
Application site folliculitis	1 (5.0)	1 (4.8)	0	0
Hand–foot–and–mouth disease	0	0	1 (5.0)	1 (5.0)
Musculoskeletal and connective tissue disorders	0	0	1 (5.0)	0
Arthralgia	0	0	1 (5.0)	0
Respiratory, thoracic, and mediastinal disorders	0	3 (14.3)	0	0
Oropharyngeal pain	0	3 (14.3)	0	0

Abbreviations: BID, twice daily; QD, once daily; TEAEs, treatment‐emergent adverse event.

^a^ Patients were only counted once per treatment per event.

In Cohort 1, five (25.0%) patients receiving crisaborole 2% QD experienced TEAEs in the crisaborole‐treated lesion and two (10.0%) patients experienced TEAEs in the vehicle‐treated lesion; four (19.0%) patients receiving crisaborole 2% BID experienced TEAEs in the crisaborole‐treated target lesion and two (9.5%) patients experienced TEAEs in the vehicle‐treated lesion. In Cohort 2, no TEAEs were reported in target lesions treated with crisaborole 2% QD, whereas TEAEs were reported in the target lesion of one (5.0%) patient receiving crisaborole 2% BID; no patient experienced TEAEs in vehicle‐treated lesions. TEAEs by system organ class and preferred term for target lesions in each cohort are provided in Table S2.

## 4 DISCUSSION

This phase 2b study assessed the efficacy and safety of two crisaborole regimens in Japanese pediatric and adult patients with mild‐to‐moderate AD. The primary efficacy endpoint was achieved with both crisaborole regimens in both cohorts: crisaborole‐treated lesions showed statistically significant reductions in TSS compared with vehicle‐treated lesions at day 15. Numerically larger decreases in TSS at day 15 were observed with crisaborole 2% BID compared with crisaborole 2% QD in both cohorts. When crisaborole and vehicle were compared in each patient, crisaborole‐treated lesions demonstrated a larger decrease in TSS at day 8 compared with vehicle‐treated lesions with both regimens in both cohorts. Decreases in TSS at day 8 were similar between crisaborole 2% QD and BID in both cohorts, indicating an early onset of improvement with both regimens in both cohorts. Furthermore, crisaborole‐treated lesions generally demonstrated greater decreases in the ISGA, peak pruritus NRS, Itch Severity Scale, and Caregiver‐Reported Itch Severity NRS compared with vehicle‐treated lesions irrespective of regimen or cohort. Improvements in ISGA and Itch Severity Scale were comparable between crisaborole 2% QD and BID at day 8 and day 15 for ISGA and day 2 to day 15 for the Itch Severity Scale; however, similar improvements in peak pruritus NRS were observed with both crisaborole regimens from day 2 to day 12 until slightly more improvement was observed with crisaborole BID compared with crisaborole 2% QD after day 13. Greater improvement in Caregiver‐Reported Itch Severity NRS was observed with crisaborole 2% BID compared with crisaborole 2% QD from day 2 to day 15. Finally, TEAEs with both crisaborole regimens were mild in both cohorts, and no patients discontinued the study. Together, these results suggest that both crisaborole regimens demonstrated efficacy and were well tolerated in Japanese pediatric and adult patients with mild‐to‐moderate AD.

Efficacy results from the current study are consistent with those of previous studies with crisaborole. Greater improvements in TSS, peak pruritus NRS, and ISGA were observed with crisaborole treatment compared with vehicle, as was observed in a phase 2a, randomized, double‐blind, vehicle‐controlled, intrapatient study of adult patients with mild‐to‐moderate AD in Canada.^21^ Similarly, the efficacy of crisaborole was consistent with that of the phase 3 CORE 1 and CORE 2 studies conducted in the USA, in which crisaborole decreased pruritus in a greater proportion of patients compared with vehicle at days 8, 15, and 29.^16^ Furthermore, consistent with findings in the phase 3 CORE 1 and CORE 2 studies,^16^ the efficacy of crisaborole was comparable across age groups in Japanese patients. Although we were unable to enroll Japanese patients aged 12–17 years in this study, efficacy of crisaborole in this age group is expected given that crisaborole was effective in non‐Japanese patients from this age group. Taken together, the efficacy of crisaborole in Japanese patients was comparable to that of non‐Japanese patients with mild‐to‐moderate AD across age groups in previous crisaborole studies.

Findings of several studies suggest that the pathophysiological features of AD are heterogeneous among racial groups.^22^, ^23^ For instance, genetic profiling of Asian and White patients with AD showed that Asian patients exhibited an AD phenotype that blended features of White patients and those of patients with psoriasis, including increased hyperplasia and higher T‐helper (Th)17 and Th22 activation.^22^ Moreover, AD lesions in Black patients tend to exhibit more lichenification compared with those of White patients.^23^ Given the paucity of AD studies in diverse patient populations,^23^ the current study addresses an important gap concerning the efficacy of crisaborole in an Asian patient population.

The safety profile of crisaborole was consistent with that of previous studies of crisaborole in patients with mild‐to‐moderate AD. The most frequently reported TEAEs in the current study included application site pain and application site pruritus, which were also reported in phase 2 studies of adolescent and adult patients with AD who received crisaborole treatment.^24^, ^25^ Japanese treatment guidelines have recommended topical corticosteroids and tacrolimus as first‐ and second‐line pharmacological therapies for AD; however, both have been associated with AEs that may promote non‐adherence.^1^, ^6^ Compared with topical corticosteroids and tacrolimus, crisaborole has a more favorable safety profile and may be more tolerable.^17^

This study had several limitations, including a small patient population. In addition, intrapatient comparisons of crisaborole and vehicle limited the interpretation of study results. Furthermore, pruritus is the most prominent symptom of AD,^26^ and the results of this study should be interpreted carefully because only target lesions were evaluated. Given that the peak pruritus NRS and Itch Severity Scale were evaluated by patients, it may have been difficult to specifically evaluate pruritus in target lesions if patients experienced pruritus in larger AD‐affected areas. In contrast, the Caregiver‐Reported Itch Severity NRS yielded objective evaluations because it was completed by caregivers who were able to focus on target lesions only. Finally, multiplicity was not adjusted for all secondary endpoints, precluding formal statistical comparisons between crisaborole and vehicle.

In conclusion, both crisaborole regimens, particularly crisaborole 2% BID, demonstrated efficacy and were well tolerated in Japanese pediatric and adult patients aged ≥2 years with mild‐to‐moderate AD. Therefore, crisaborole, 2%, may be a viable treatment option for Japanese patients with mild‐to‐moderate AD.

## CONFLICT OF INTEREST

K. Fujita and M. Yoshida are employees of Pfizer R&D Japan, and K. Fujita is a stockholder of Pfizer Inc. M. Yagi has received research grants from Pfizer R&D Japan. S. Moriwaki has nothing to disclose. D. Graham is an employee and stockholder of Pfizer Inc.

## Supporting information

Table S1‐S2

## Data Availability

Upon request, and subject to certain criteria, conditions and exceptions (see https://www.pfizer.com/science/clinical‐trials/trial‐data‐and‐results for more information), Pfizer will provide access to individual de‐identified participant data from Pfizer‐sponsored global interventional clinical studies conducted for medicines, vaccines and medical devices (1) for indications that have been approved in the US and/or EU or (2) in programs that have been terminated (i.e., development for all indications has been discontinued). Pfizer will also consider requests for the protocol, data dictionary, and statistical analysis plan. Data may be requested from Pfizer trials 24 months after study completion. The de‐identified participant data will be made available to researchers whose proposals meet the research criteria and other conditions, and for which an exception does not apply, via a secure portal. To gain access, data requestors must enter into a data access agreement with Pfizer.
