# Supplementary material for: A phase 2b, randomized, double‐blind, multicenter, vehicle‐controlled study to assess the efficacy and safety of two crisaborole regimens in Japanese patients aged 2 years and older with mild‐to‐moderate atopic dermatitis
Source: J Dermatol. 2021 Aug 26;48(11):1640–51. doi: 10.1111/1346-8138.16120 (PMC9292399; doi:10.1111/1346-8138.16120)
Supplement: Supplementary file 1 — Table S1‐S2 [file JDE-48-1640-s001.docx]

**A phase 2b, randomized, double-blind, multicenter, vehicle-controlled study to assess the efficacy and safety of two crisaborole regimens in Japanese patients aged ≥2 years with mild-to-moderate atopic dermatitis**

**Authors:** Kayo Fujita,^1^ Michio Yagi,^2^ Shinichi Moriwaki,^3^ Mizuki Yoshida,^4^ Daniela Graham^5^

**Affiliations:** ^1^Clinical Research, Pfizer R&D Japan, Tokyo, Japan; ^2^OPHAC Hospital, Osaka, Japan; ^3^Department of Dermatology, School of Medicine, Osaka Medical and Pharmaceutical University, Osaka, Japan; ^4^Clinical Statistics, Pfizer R&D Japan, Tokyo, Japan; ^5^Pfizer Inc., Groton, CT, USA

**Short title:** Crisaborole treatment of AD in Japan

**Corresponding author:**

Kayo Fujita, PhD

Pfizer R&D Japan

3-22-7, Yoyogi, Shibuya-ku

Tokyo 151-8589 Japan

Phone: +81-80-5880-4719

Email: kayo.fujita@pfizer.com

**SUPPORTING INFORMATION**

**Table S1.** Target lesion baseline characteristics (full analysis set)

|  | **Cohort 1 (aged ≥12 years)** | | | | **Cohort 2 (aged 2-11 years)** | | | |
| --- | --- | --- | --- | --- | --- | --- | --- | --- |
|  | **QD regimen**  ***n* = 20** | | **BID regimen**  ***n* = 21** | | **QD regimen**  ***n* = 20** | | **BID regimen**  ***n* = 20** | |
|  | **Crisaborole 2%** | **Vehicle** | **Crisaborole 2%** | **Vehicle** | **Crisaborole 2%** | **Vehicle** | **Crisaborole 2%** | **Vehicle** |
| **TSS** |  |  |  |  |  |  |  |  |
| *n* | 20 | 20 | 21 | 21 | 20 | 20 | 20 | 20 |
| Mean (SD) | 7.7 (1.6) | 7.5 (2.0) | 7.1 (2.1) | 7.4 (2.2) | 6.2 (1.7) | 6.5 (1.7) | 7.1 (2.0) | 7.3 (2.0) |
| Median (range) | 8.0 (4–11) | 7.5 (4–12) | 6.0 (3–11) | 7.0 (4–11) | 6.0 (4–9) | 6.0 (4–9) | 8.0 (4–10) | 7.5 (3–10) |
| **Peak Pruritus NRS** |  |  |  |  |  |  |  |  |
| *n* | 20 | 20 | 21 | 21 | **–** | **–** | **–** | **–** |
| Mean (SD) | 5.3 (2.7) | 5.3 (2.5) | 5.1 (2.1) | 5.3 (2.0) | **–** | **–** | **–** | **–** |
| Median (range) | 6.0 (1–9) | 5.5 (2–9) | 5.0 (1–9) | 5.0 (2–9) | **–** | **–** | **–** | **–** |
| **Itch Severity Scale** |  |  |  |  |  |  |  |  |
| *n* | **–** | **–** | **–** | **–** | 16 | 16 | 14 | 14 |
| Mean (SD) | **–** | **–** | **–** | **–** | 1.6 (1.3) | 1.8 (1.3) | 1.9 (1.2) | 1.6 (1.3) |
| Median (range) | **–** | **–** | **–** | **–** | 1.0 (0–4) | 2.0 (0–4) | 2.0 (0–3) | 1.5 (0–4) |
| **Caregiver-Reported Itch Severity NRS** |  |  |  |  |  |  |  |  |
| *n* | **–** | **–** | **–** | **–** | 20 | 20 | 20 | 20 |
| Mean (SD) | **–** | **–** | **–** | **–** | 4.7 (2.5) | 5.0 (2.2) | 5.5 (2.5) | 5.1 (2.5) |
| Median (range) | **–** | **–** | **–** | **–** | 4.5 (1–10) | 5.0 (1–9) | 5.5 (1–9) | 5.0 (0–9) |

BID, twice daily; NRS, numeric rating scale; QD, once daily; SD, standard deviation; TSS, total sign score.

**Table S2.** TEAEs by System Organ Class and Preferred Term in target lesions of Japanese pediatric and adult patients receiving crisaborole 2% or vehicle ^†^ (safety analysis set)

| **Cohort 1 (aged ≥12 years)** | | | | |
| --- | --- | --- | --- | --- |
|  | **QD regimen**  ***n* = 20** | | **BID regimen**  ***n* = 21** | |
| ***n*** **(%)** | **Crisaborole 2%** | **Vehicle** | **Crisaborole 2%** | **Vehicle** |
| **Any adverse event** | 5 (25.0) | 2 (10.0) | 4 (19.0) | 2 (9.5) |
| **General Disorders and Administration Site Conditions** | 4 (20.0) | 2 (10.0) | 4 (19.0) | 2 (9.5) |
| Application site irritation | 2 (10.0) | 1 (5.0) | 4 (19.0) | 1 (4.8) |
| Application site pruritus | 2 (10.0) | 2 (10.0) | 1 (4.8) | 1 (4.8) |
| Application site pain | 1 (5.0) | 0 | 1 (4.8) | 0 |
| Application site coldness | 0 | 0 | 0 | 1 (4.8) |
| **Infections and Infestations** | 1 (5.0) | 0 | 1 (4.8) | 0 |
| Application site folliculitis | 1 (5.0) | 0 | 1 (4.8) | 0 |
| **Cohort 2 (aged 2-11 years)** | | | | |
|  | **QD regimen**  ***n* = 20** | | **BID regimen**  ***n* = 20** | |
| ***n* (%)** | **Crisaborole 2%** | **Vehicle** | **Crisaborole 2%** | **Vehicle** |
| **Any adverse event** | 0 | 0 | 1 (5.0) | 0 |
| **General Disorders and Administration Site Conditions** | 0 | 0 | 1 (5.0) | 0 |
| Application site irritation | 0 | 0 | 0 | 0 |
| Application site pruritus | 0 | 0 | 1 (5.0) | 0 |
| Application site pain | 0 | 0 | 1 (5.0) | 0 |
| Application site coldness | 0 | 0 | 0 | 0 |
| **Infections and Infestations** | 0 | 0 | 0 | 0 |
| Application site folliculitis | 0 | 0 | 0 | 0 |

**^†^**Patients were only counted once per treatment per event.

BID, twice daily; QD, once daily; TEAE, treatment-emergent adverse event.
